# Round the Hospitals

**Published:** 1916-08-12

**Authors:** 


### ROUND THE HOSPITALS.

Matrons, sisters, and nurses who are fortunate enough to be off duty or away on holiday, and have full opportunity to enjoy the sunshine and the warmth that have come at last to these islands, should feel grateful to Miss Rundle and her staff for remaining in the office and working continuously. There can be no holiday for them until the Register is well forward, and every member of the profession who has the wit and energy to look after her own interests should help by sending in her application and papers for registration without a day's delay to the Secretary, the College of Nursing, 6 Vere Street, Oxford Street, W. There is none too much time for those nurses who are ambitious to be amongst the first 7,000 pioneers of the Royal College of Nursing. With a view to meeting the convenience of matrons, sisters, and nurses on active service, the Council of the College has waived the application of candidates for registration to forward with their application forms copies of certificates they hold from their nurse-training schools. These certificates, however, will be required later that they may be filed. This wise concession will prove of great benefit to nurses working abroad, and all serving in military hospitals at home will be glad to be relieved from the difficulty of procuring their certificates, which they may not have with them at the moment. We hope every member of the profession will realise the many details which have to be attended to and adjusted, to enable the formalities of registration to be proceeded with rapidly. Everything is being pushed on as quickly as possible, and directly the necessary alterations in the Articles and other matters have been effected, the present delays will cease and everything should proceed rapidly. Everyone who has sent in their papers will facilitate the work by exercising patience in case of delay, which will be unavoidable, at least until after August 20, 1916.

We regret to hear that the solicitor of the Royal British Nurses' Association has been confined to bed as the result of a motor accident, from which we are glad to hear he is now recovering. This accident and the absence of one or more of the active leaders of the Registrationists may, it is thought, cause delay in arriving at the final decision on an agreed Bill. It would be remarkable should the passing of this Registration Bill in the autumn session of Parliament this year be delayed, or rendered impossible, by the absence on holiday of the chief advocate of nurses registration, and we can only suppose that the story current in nursing circles is nothing but a rumour, which lacks substantial foundation. We hear authoritatively, in any case, that the Registration Bill will be proceeded with after August 20, and that all who wish to be amongst the 7,000 pioneers of the College and Bill should prepare their papers and lodge them with Miss Rundle at 6 Vere Street, W., not later than the 18th instant, or they may be crowded out (see p. 459).

Some wild statements have been recently made in regard to the number of so-called trained nurses who have received their training in Poor-Law infirmaries. Mr. H. Dawes, for instance, on behalf of the Executive Committee, at the annual meeting of the National Association of Masters and Matrons of Poor-Law Infirmaries, is reported to have said: "It had been ascertained that 75 per cent. of the nurses in Great Britain were Poor-Law trained nurses." This statement is on the face of it absurd, and neither Mr. Dawes nor the Executive Committee of the National Association of Poor-Law Institutions have produced evidence upon which these figures are based. They cannot in any case relate to fully-trained nurses, as the official facts available demonstrate conclusively. According to the latest returns received from the authorities of the Training Schools in England and Wales, there are some 5,500 women undergoing training in the voluntary hospitals and nurse-training schools, and a further 1,000 working in smaller institutions where the training, to say the least, is not adequate. The number of women returned as actual probationers undergoing training in Poor-Law infirmaries in England and Wales is slightly over 4,000, against upwards of 5,500 probationers in the nurse-training schools and hospitals in England and Wales. This shows that the actual

**MATRONS' AND SISTERS' DEPARTMENT—(*continued*).**

number of nurses in training at the Poor-Law infirmaries where a recognised system of training is organised, instead of being, as stated, 75 per cent. of the nurses, is in fact, in England and Wales, considerably less than one-half of the 9,600 women now in course of being trained in these countries—that is to say, rather more than 4,000 probationers only are being trained in Poor-Law infirmaries. In Scotland, whereas there are some 1,000 probationers undergoing training in recognised hospitals with training schools, considerably less than 400 other women are being trained in Scotch Poor-Law infirmaries. We might add to the figures by including hospitals with 50 to 100 beds, where there are some 1,000 women employed as probationers, but they cannot justly claim to be efficiently trained, and we therefore exclude them, or the case against Mr. Dawes's figures would be even more overwhelming.

Ever since nurses in the Poor-Law Service in self-defence contracted themselves out of the pension scheme for Poor-Law officers, they in practice have dissociated themselves from the National Association. This may account for the extraordinary inaccuracy of the claim put forward by Mr. H. Dawes on behalf of his Executive Committee in regard to the number of Poor-Law nurses. In order to arrive at these preposterous figures, Mr. Dawes must have included, we presume, every woman employed anywhere in an institution, or engaged in work, under the Poor Law, which in any way touches the imbecile, the infirm, the aged, the chronically disabled, the children, i.e. every type of person under the Poor-Law system where women attendants of any description are engaged. It is just to Mr. H. Dawes and his Executive Committee to add that he explained that there were other nurses in the country besides hospital nurses, but he gave no definition of the term "nurse." Whatever definition may be given of the 75 per cent. of so-called nurses, it is quite clear that they cannot for the most part be certificated trained nurses, or trained nurses at all in any proper meaning of the word. We venture in this connection to call the attention of the Right Hon. Walter Long and the officials of the Local Government Board to the figures here referred to, in the hope that steps may be taken to prevent the circulation in future of erroneous statistics relating to Poor-Law matters of the misleading type of those to which we have felt it our duty to here call attention.

Trained hospital nurses are specifically mentioned among the "poor gentlewomen honestly striving to earn their own livelihood" who are to benefit by the establishment of a convalescent home under the will of Mr. Frederick Andrew, a Lincoln solicitor. Mr. Andrew desired that the home, which is to bear his name, should be situated within 100 miles of London, and might be made "a means of restful happiness and real use by restoring to health many generations of a hitherto much-neglected though very deserving class." His instructions are that the house is to be of a picturesque elevation, constructed within and without of the best materials, and "seated in an extensive and old-world garden of old-fashioned flowers, and that it be refined and beautified from time to time as the funds might permit." It is estimated that £100,000 will be available for the purposes of this trust.

